# The Tilted Self: Visuo-Graviceptive Mismatch in the Full-Body Illusion

**DOI:** 10.3389/fneur.2019.00436

**Published:** 2019-05-08

**Authors:** Carla Thür, Marte Roel Lesur, Christopher J. Bockisch, Christophe Lopez, Bigna Lenggenhager

**Affiliations:** ^1^Department of Psychology, University of Zurich, Zurich, Switzerland; ^2^Department of Neurology, University Hospital Zurich, Zurich, Switzerland; ^3^Department of Otorhinolaryngology, University Hospital Zurich, Zurich, Switzerland; ^4^Department of Ophthalmology, University Hospital Zurich, Zurich, Switzerland; ^5^Aix Marseille University, CNRS, LNSC, FR3C, Marseille, France

**Keywords:** full-body illusion, vestibular system, multisensory integration, out-of-body experience, bodily orientation, haptic vertical

## Abstract

The bodily self is a fundamental part of human self-consciousness and relies on online multimodal information and prior beliefs about one's own body. While the contribution of the vestibular system in this process remains under-investigated, it has been theorized to be important. The present experiment investigates the influence of conflicting gravity-related visual and bodily information on the sense of a body and, vice versa, the influence of altered embodiment on verticality and own-body orientation perception. In a full-body illusion setup, participants saw in a head-mounted display a projection of their own body 2 m in front of them, on which they saw a tactile stimulation on their back displayed either synchronously or asynchronously. By tilting the seen body to one side, an additional visuo-graviceptive conflict about the body orientation was created. Self-identification with the seen body was measured explicitly with a questionnaire and implicitly with skin temperature. As measures of orientation with respect to gravity, we assessed subjective haptic vertical and the haptic body orientation. Finally, we measured the individual visual field dependence using the rod-and-frame test. The results show a decrease in self-identification during the additional visuo-graviceptive conflict, but no modulation of perceived verticality or subjective body orientation. Furthermore, explorative analyses suggest a stimulation-dependent modulation of the perceived body orientation in individuals with a strong visual field dependence only. The results suggest a mutual interaction of graviceptive and other sensory signals and the individual's weighting style in defining our sense of a bodily self.

## Introduction

The continuous representation of the own body and its relation to the external world is an important part of the daily experience of our self. Such representations are thought to be based on a probabilistic integration of body signals from various sensory systems and prior beliefs about the body (1). The sense of our bodily self, which includes the feeling of body ownership, self-location, and first-person perspective (2), is thus surprisingly plastic and constantly updated by the current sensory signaling. Over the last years, experimental setups that systematically present synchronous but conflicting inputs from different sensory modalities have been developed to alter and study such updating processes.

In the seminal rubber hand illusion (3), synchronous but conflicting information about where a tactile event is seen (on a rubber hand in front of the participant) and where it is felt (on the real hand of the participant, hidden from sight) induces a temporary illusory sense of ownership over the rubber hand. Such a subjective change in the bodily self corroborates various perceptual and physiological measures, such as drops in skin temperature recorded on the participant's hand (4) [but see Ref. (5) for a critical view]. In this setting, the spatially conflicting information is thus presented in a body-centered reference frame (i.e., the visual and bodily information about the tactile event concerning the hand locations differs in relation to the rest of the body). The information about the position and orientation of the body in space with respect to gravity remains stable. Related illusions have been developed to investigate more global body representations (6), in which the conflicting information is spatially presented in an allocentric reference frame (i.e., the position of the full body in space). Consequently, in these full-body illusions, vestibular and other graviceptive (proprioceptive and interoceptive) systems might play an important role, as they both encode self-orientation in relation to gravity (7, 8). Recent theoretical and empirical work shows that vestibular signals importantly contribute to higher-level space and body perception (9–11) and bodily self-awareness [see, e.g., Refs. (12, 13) for extensive reviews]. Yet, very few studies have directly investigated the mutual interactions between visuo-graviceptive conflicts and bodily self-consciousness [for exceptions, see Refs. (14–18)].

**Figure 1:**
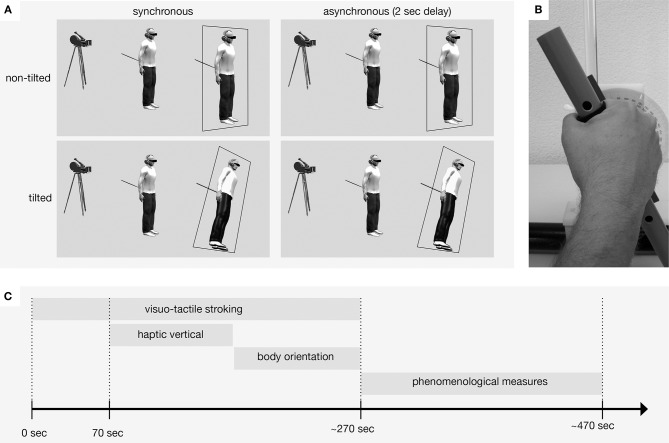
Experimental setup and procedure. **(A)** Illustration of the four experimental conditions: Participants were seeing on the HMD their own body in front of them either in an upright position or in a tilted position, and the stroking on the back was shown either synchronously or asynchronously with the felt stroking. **(B)** Device that was used to measure subjective haptic vertical and subjective body orientation. **(C)** Procedure of one condition. First, the stroking was applied to induce the illusion, then while still experiencing the multisensory stimulation, participants completed the haptic vertical and the subjective body orientation task. Afterwards, the stimulation was halted and participants filled out a questionnaire on the phenomenological aspects of the illusion.

Here, we set out to test how conflicting visual and graviceptive signals in a full-body illusion and resulting perceptual changes might affect perceived body and gravity orientation. For this, we created a full-body illusion, in which participants see a video of their own body in a head-mounted display (HMD), as if it were projected 2 m in front of them [for details, see Ref. (6) and Figure 1A]. Tactile stroking was applied to their back while participants saw their own back in front of them being touched synchronously (to increase self-identification with the seen body) or asynchronously (as a control condition) to the felt touch. Importantly, to additionally create a visuo-graviceptive conflict about the body orientation in space, we displayed the seen body and its surroundings in an orientation that is either congruent with the participant's body orientation (upright, 0°) or incongruent with the participant's body orientation (tilted 30° counter-clockwise relative to gravity).

We measured self-identification with the seen body using questionnaires (3, 6) and skin temperature. Previous studies showed that skin temperature drops during illusory self-identification and might thus be an implicit measure of self-identification with the seen body (16, 19). To test our main hypothesis that illusory self-identification with a tilted body changes the perception of gravity and/or the perceived own-body orientation in space, we measured subjective haptic vertical and subjective body orientation.

In line with previous literature, we expect synchronous visuo-tactile stroking to increase self-identification with the seen body, with an associated decrease in skin temperature (19). During synchronous stroking in the tilted condition, we expect that self-identification with a seen tilted body will bias vertical perception and own-body orientation perception. It is well-known that actual body tilt in the roll plane changes visual vertical perception, leading to an A-effect (i.e., under compensation for large body tilts) or an E-effect (i.e., over compensation for small body tilts) (20). For haptic vertical perception of small body tilts, Schuler et al. (21) found a slight overcompensation in subjective haptic vertical judgment. This result is in line with earlier findings, which found a slight overcompensation up to 90° body tilt (22) and an overcompensation of 5° at a 35° body tilt (23). If participants identify with the seen 30°-tilted body, we expect them to overcompensate their haptic vertical judgment and to align their body orientation perception in the direction of the tilted body shown in the HMD.

As sensory weighting strategies have been previously shown to influence self-location in a variant of the full-body illusion (9, 13), we expect that individuals with a stronger visual field dependence, as measured by the rod-and-frame test, will show a stronger illusion and a more strongly altered verticality judgment.

Alternatively, the additional mismatch between visual and graviceptive information in the tilted conditions might also decrease illusory embodiment. Previous research testing the limits of plasticity in the rubber hand illusion paradigm showed that an additional spatial misalignment between the real and the rubber hand (a rotation next to the shift) decreased the illusion (24), even for small rotations (25).

## Materials and Methods

### Participants

Forty participants were recruited, but three participants were excluded for technical reasons and two due to cybersickness. The remaining sample included 35 healthy, right-handed participants (aged 18–41 years, mean ± SD: 22.9 ± 4.3 years, 10 males). All participants were naive to the study aims, had normal or corrected-to-normal vision, and declared no history of psychiatric, neurological, or vestibular diseases. They were recruited through the psychology mailing list of the University of Zurich and received study credits. The protocol was approved by the Ethics Committee of the Faculty of Arts and Social Sciences at the University of Zurich (Approval number 17.12.15), and all participants gave written informed consent prior to inclusion in the study.

### Experimental Procedures

#### Familiarization With the Protocol

Informed consent was obtained and demographic data were gathered. Following this, participants were familiarized with the procedures and the haptic device, and completed a practice trial. After making sure that they understood the task, the experiment started.

#### The Full-Body Illusion

##### Procedures

To induce the full-body illusion, we adapted the paradigm [detailed in Ref. (6)]. Participants were instructed to stand straight and not move during the experiment. In order to verify how much the participants moved, the head movements where tracked using the HMD (Oculus Rift; Oculus VR, Irvine, CA, USA) that was used for the visual presentation [see Supplementary Online Material for methods and results (see Table S1) of the head tracking]. The participant's body was filmed from behind at a 2 m distance with a Logitech c930e webcam (Logitech, Lausanne, Switzerland). The video was mapped to a digital 3D object, approximately matching the distortion of the webcam, and then projected on an HMD, using software developed in Unity 2017.3.0. The participants were touched on their back with a wooden stick. An experimenter, who was located outside the visual field of the camera, applied the touch manually. Stroking was applied on the back in an unpredictable way (which has been suggested to increase the illusion), at a rate of about one stroke per second. Four conditions were designed; the felt touch could be seen either synchronously (with an intrinsic system delay of approximately 135 ms measured at a rate of 240 Hz, thus below detectable threshold) or asynchronously (a constant delay of 2 s in addition to the intrinsic delay) on the seen body, which could be seen either upright or tilted with the virtual environment by 30° to the left (Figure 1A). Each condition was presented once in a counterbalanced order between participants. Each of the four trials (see Figure 1C) consisted of a stimulation period of 70 s, in which participants were instructed to focus on the visual scene and the tactile stimulation. Then, eight consecutive beeps instructed the participants to make judgments about their haptic vertical (see below for details). After that, another eight consecutive beeps instructed the participants to judge their own-body orientation with respect to gravity (see below for details). These two tasks took about 100 s, each depending on speed of answer. Importantly, the stroking and the visual input continued throughout these tasks. After that, the stroking was stopped, and participants answered a questionnaire shown on a black background on the HMD before the next trial started. One trial took ~5 min.

##### Measures

*Subjective haptic vertical and body orientation*. After 70 s of stroking, an auditory signal instructed participants to start with the verticality judgment. A motor-driven haptic vertical device (see Figure 1B) was used for the judgment [for further information about the device, see Ref. (26)]. The device was fixed in front of the participants at a height of 120 cm, and the rod was calibrated to 0° before every experiment. Participants were instructed to align the rod with the perceived direction of gravity (“subjective haptic vertical,” eight times) and thereafter with their foot-head axis (“subjective body orientation,” eight times). The rod position was sampled at 200 Hz using Labview (National Instruments). After each judgment, the rod automatically moved to a new random position between ±75° from upright.

*Questionnaire*. After the haptic judgment tasks, a German version of a questionnaire adapted to a previous full-body illusion questionnaire (6), including additional items about the vestibular perception, was presented in the HMD. For clarity, the questionnaire was subdivided into four categories based on the content of the questions (see Table 1). The questions were answered on a visual analog scale ranging from 0 (“not at all”) to 1 (“very strongly”) by controlling a continuously moving cursor with head movements. Once the cursor was at the selected position on the visual analogue scale (VAS) scale, participants answered by selected with the cursor an OK button and the answer was recorded using Unity. This way, participants did not have to remove the HMD between the conditions or use an extra controller.

**Table 1:** The table shows the results of the Friedman tests for all dependent variables and *post hoc* Wilcoxon comparisons for the significant effects.

**Results**	**Friedman-test**		**NT (S vs. AS) (Illusion-effect upright)**	**T(S vs. AS) (Illusion-effect tilt)**	**S (NT vs. T) (Tilt-effect synchronous)**	**AS (NT vs. T) (Tilt-effect asynchronous)**
**QUESTIONNAIRE**						
***Ownership related questions***						
Q1: …You were looking at someone else?	*x*^2^ = 14.7	*p =* 0.002^*^	*p =* 0.003^*^	*p =* 0.31	*p =* 0.002^*^	*p =* 0.94
Q2: …You had more than one body	*x*2 = 13.5	*p =* 0.004^*^	*p =* 0.002^*^	*p =* 0.14	*p =* 0.004^*^	*p =* 0.16
Q6: …The body you saw was your body?	*x*2 = 12.2	*p =* 0.007'	*p* < 0.001^*^	*p =* 0.20	*p =* 0.10	*p =* 0.40
***Disembodiment related*** **questions**						
Q5: …You were located at two places?	*x* ^2^ = 14.1	*p* = 0.003^*^	*p* = 0.011^*^	*p =* 0.80	*p =* 0.002^*^	*p =* 0.80
Q8: …You were separated from your body (as if yourself and body were localized at two different places)?	*x*2 = 13.1	*p =* 0.005^*^	*p =* 0.003^*^	*p =* 0.33	*p =* 0.039	*p =* 0.63
***Referral of touch related question***						
Q7: …The seen touch was the one you felt?	*x*^2^ = 31.6	*p* < 0.001^*^	*p* < 0.001^*^	*p* < 0.001^*^	*p =* 0.16	*p =* 0.79
***Balance, stability and orientation related*** **questions**						
Q3: …You were swaying back and forth?	*x*^2^ = 8.0	*p =* 0.047^*^	*p* = 0.15	*p =* 0.66	*p* = 0.02	*p =* 0.70
Q4: …You lost balance?	*x*^2^ = 2.6	*p* = 0.47				
Q9: …You were tilted to left or right?	*x*^2^ = 10.7	*p =* 0.014^*^	*p* = 0.96	*p =* 0.012^*^	*p =* 0.15	*p* < 0.001^*^
Q10: …You were floating?	*x*^2^ = 1.3	*p* = 0.72				
Q11: …You felt sick?	*x*^2^ = 2.5	*p =* 0.48				
**VERTICALITY**						
Subjective haptic vertical	*x*^2^ = 4.0	*p* = 0.26				
Subjective body orientation	*x*2 = 24.8	*p* < 0.001^*^	*p =* 0.88	*p =* 0.23	*p* < 0.001^*^	*p* < 0.001^*^
**SKIN TEMPERATURE**						
Electrode neck	*x*^2^ = 2.65	*p =* 0.45				
Electrode collarbone	*x* ^2^ = 1.66	*p =* 0.65				
Electrode left arm	*x*2 = 2.09	*p* = 0.55				

* *indicates significance level. For Friedman tests, it was set to 0.05, and for the post hoc tests, it was set to p = 0.0125 according to the Bonferroni correction. NT, non-tilted; T, tilted; S, synchronous stroking; AS, asynchronous stroking*.

*Skin temperature*. During the stroking period, we continuously measured skin temperature with a sampling rate of 2 Hz with an HH309A Data Logger Thermometer (Omega, Stamford, CT, USA), through four sensors (16). Two sensors were placed in camera field of view, i.e., one at the back of the neck and one at the back of the left arm. A third sensor was placed on the collarbone, which was not visible in the HMD, as previous studies indicated a drop in temperature only for seen body parts (16). A forth measured changes in the room temperature.

#### The Rod-And-Frame Test

At the end of the experiment, visual field dependence was measured with the rod-and-frame test (27). MATLAB R2017b was used for presentation of the rod-and-frame test and for recording responses. A white dotted line (8.6 cm in length) was presented on the screen. Participants were asked to adjust the line inside a square to a vertical position. The initial position in which the line was shown was either tilted counter-clockwise (four trials) or clockwise (four trials) at a randomly chosen angle (in the range of ±4° with respect to the gravitational vertical). The frame was tilted 20° clockwise eight times and was upright eight times. The order of the two conditions was counterbalanced. The room was completely dark and a round frame covered the screen edges, so the participants could not refer to vertical objects around them.

### Data Processing and Statistical Analysis

#### Preprocessing

##### Subjective haptic vertical and body orientation

For each of these two measures, we calculated the mean and standard deviation of the eight repetitions. One participant was excluded for technical reasons.

##### Skin temperature

For each condition, the mean value of the first 1.5 s (three measure points) was used to calculate a baseline. The baseline-corrected values of the data points from the following 70 s were then averaged. Finally, the baseline-corrected room temperature was subtracted from all temperature averages. Four participants were excluded for technical reasons.

*The rod-and-frame test* was analyzed by calculating the mean of the eight trials for each condition (tilted frame/upright frame). A hierarchical cluster analysis was used to form two groups based on their visual field dependence/independence (28). For this, Ward's aggregation method was used (SPSS 24), and the Euclidean distance between participants was calculated based on the values of the tilted and the upright frame and a hierarchical tree was formed. The tree was divided at the maximum of dissimilarity into two clusters of visual-field-dependent and visual-field-independent participants. Two participants were excluded for technical reasons.

#### Statistics

Statistical analysis was performed using R 3.5.0 GUI 1.70. First, the Shapiro–Wilk test revealed non-normally distributed data for most of the dependent variables. We thus used non-parametrical tests, by first testing the effect of Condition (synchronous/tilted, asynchronous/tilted, synchronous/non-tilted, asynchronous/non-tilted) using Friedman tests. For significant effects only, we used Wilcoxon tests to compare the effect of Synchrony (synchronous vs. asynchronous visuo-tactile stroking) for the tilted and non-tilted conditions, separately. In addition, we compared the effect of Tilt (non-tilted vs. tilted) separately for the synchronous and asynchronous conditions. We used a Bonferroni-corrected *p* value to account for the number of comparisons for each dependent variable (*n* = 4, corrected *p*-value: 0.0125).

To test the effect of visual field dependence on the variables of interest (i.e., the relative differences between synchronous and asynchronous stroking in the two different tilts, and the relative differences between the two different tilts in both types of stroking), we calculated the relative values by subtracting the asynchronous from the synchronous conditions and the non-tilted from the tilted conditions. To compare relative dependent variables between visual-field-dependent and -independent participants, we used Mann-Whitney *U*-tests.

## Results

### Explicit Measures of the Illusion: Questionnaire

**Figure 2:**
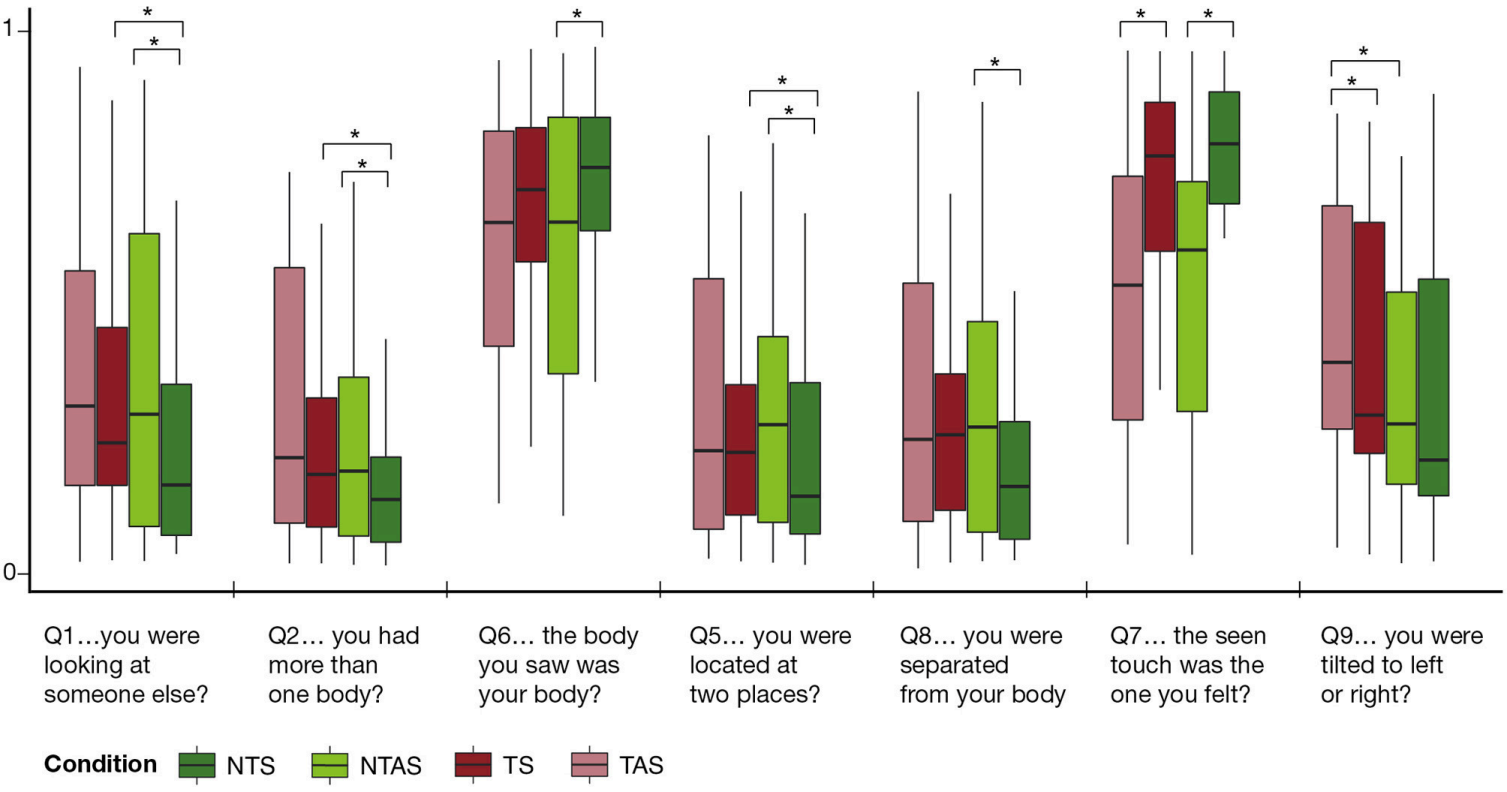
Results of the questionnaire. For all items that revealed a significant effect of Condition, the medians and interquartile ranges for each condition are plotted. Significance bars refer to the results of the Bonferroni-corrected *post hoc* Wilcoxon tests. ^*^ indicates a significant effect. NT, non-tilted; T, tilted; S, synchronous stroking; AS, asynchronous stroking.

Table 1 shows all questionnaire items and results of the Friedman test, as well as *post hoc* comparisons for the significant effects. The Friedman test revealed a significant effect of Condition for questions related to ownership (Q1, Q2, and Q6), disembodiment (Q5 and Q8), touch (Q7), and balance and orientation-related questions (Q3 and Q9) (*p* < 0.047, χ^2^ > 8.0, see Figure 2). The Friedman test was not significant for the other vestibular-related questions Q4, Q10, and Q11 (*p* > 0.47, χ^2^ < 2.6).

#### Embodiment-Related Questions

The *post hoc* comparisons (see Figure 2) showed that in the non-tilted condition, ownership was higher in the synchronous than in the asynchronous condition. This was evidenced by a stronger feeling that the seen body was felt as their own (Q6), a lower sensation of looking at someone else (Q1), and a feeling that they had more than one body (Q2). Similarly, the referred sensation of touch (Q7) was stronger in the synchronous than in the asynchronous condition, and the feeling of disembodiment (Q5 and Q8) was stronger in the asynchronous than in the synchronous condition.

For the tilted conditions, of all these effects, only the one for referral of touch (Q7) was significant. This is further corroborated by the significant difference in the synchronous conditions between non-tilted and tilted for questions Q1, Q2, and Q5.

#### Body Orientation and Stability-Related Questions

The only item that revealed significant differences in the *post hoc* comparison was the question whether participants felt tilted (Q9) and suggested that they felt more tilted during asynchronous stroking than during synchronous stroking.

### Implicit Measures: Skin Temperature, Verticality, and Subjective Body Orientation Judgment

#### Skin Temperature

The Friedman test revealed no significant effect of Condition on baseline-corrected skin temperature measured on the neck (χ^2^ = 2.65, *p* = 0.45), collarbone (χ^2^ = 1.66, *p* = 0.65), and left arm (χ^2^ = 2.09, *p* = 0.55).

#### Subjective Haptic Vertical

The Friedman test did not reveal a significant effect of Condition on the subjective haptic vertical (χ^2^ = 4.0, *p* = 0.26). A further analysis of the effect of Condition on the standard deviation of the haptic vertical was also not significant (χ^2^ = 2.3, *p* = 0.5).

#### Subjective Body Orientation

The Friedman test showed a significant effect of Condition (χ^2^ = 24.8, *p* < 0.001). Table 1 shows the significant *post hoc* comparisons. The perceived own-body orientation was significantly more tilted to the left (thus in the direction of the seen body) in the tilted compared to the non-tilted conditions, for both synchronous (*p* < 0.001) and asynchronous (*p* < 0.001) visuo-tactile stroking. The analysis of the effect of Condition on the standard deviation of subjective body orientation was not significant (χ^2^ = 7.55, *p* = 0.06).

### Modulatory Effect of Visual Field Dependence

Hierarchical clustering revealed a group of visual-field-dependent participants (*n* = 13, mean value non-tilted = −0.05°, mean value tilted = 1.55°) and a group of visual-field-independent participants (*n* = 20, mean value non-tilted = −0.13°, mean value tilted = −0.30°). There was a significant effect of Group for both the relative subjective body orientation and the relative subjective haptic vertical.

#### Subjective Haptic Vertical

A Mann-Whitney test indicated that visual-field-dependent participants aligned their subjective haptic vertical more to the seen body in the tilted condition compared to the non-tilted condition during synchronous stroking (Mdn = −1.70°) than did visual-field-independent participants (Mdn = −0.10°, *U* = 186, *p* = 0.02).

#### Subjective Body Orientation

**Figure 3:**
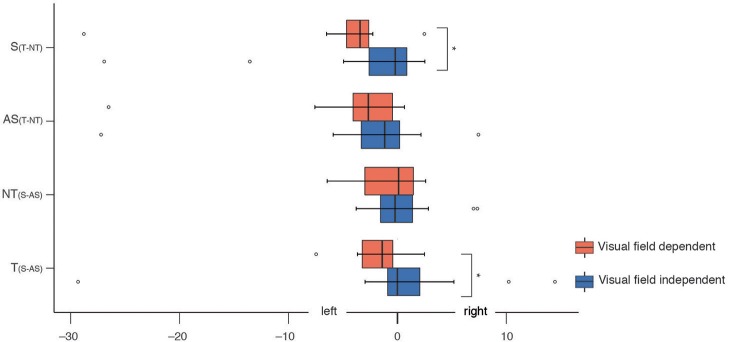
Results of the effect of visual dependence on haptic subjective body orientation. Medians and interquartile ranges for the relative values of the subjective body orientation are plotted for the visual dependent group and the not visual dependent group. Significance bars refer to the results of the Mann-Whitney tests. ^*^indicates significant effects. S_(T−NT)_ indicates the relative values for the synchronous tilted as compared to the synchronous non-tilted condition. AS_(T−NT)_ indicates the relative values for the asynchronous tilted as compared to the asynchronous non-tilted condition. NT_(S−AS)_ indicates the relative values for the non-tilted synchronous as compared to the non-tilted asynchronous condition. T_(S−AS)_ indicates the relative values for the tilted synchronous as compared to the tilted asynchronous condition.

A Mann-Whitney test indicated that in the tilted condition, visual-field-dependent participants aligned their subjective body orientation more toward the seen body during synchronous relative to asynchronous visuo-tactile stroking (Mdn = −1.37°) than did visual-field-independent participants (Mdn = −0.03°, *U* = 183, *p* = 0.02). Similarly, during synchronous visuo-tactile stroking, visual-field-dependent participants aligned their subjective body orientation more strongly in the direction of the seen body in the tilted relative to the non-tilted condition (Mdn = −3.37°) than did visual-field-independent participants (Mdn = −0.50°, *U* = 181, *p* = 0.03, see Figure 3).

## Discussion

This study investigates illusory self-identification and self-orientation perception in a multisensory stimulation paradigm. Participants saw in an HMD a projection of their own body 2 m in front of them and felt tactile stimulation on their back either synchronously or asynchronously to the seen touch [full-body illusion setup (6)]. We exposed participants to an additional visuo-graviceptive conflict by presenting them with the projected body in an orientation that was congruent (upright) or incongruent (tilted) with the participant's actual upright body orientation. The study revealed three main findings. First, while we replicated self-identification with the seen body during synchronous stroking on a phenomenological level, questionnaire data suggest, in line with the alternative hypothesis, a decrease in the illusion strength during additional visuo-graviceptive conflict about the body orientation in space with respect to gravity. Second, we did not find a modulation of the perceived vertical and own-body orientation for the tilted body by synchrony of the stroking at the group level. Third, an analysis accounting for idiosyncratic strategies in multisensory integration (29) suggests a stimulation-dependent modulation of the perceived body orientation only in individuals with a visual field dependence.

### Effect of Visual-Otolithic Conflict on the Full-Body Illusion

Our data show that the full-body illusion, as determined by explicit measures, is attenuated by the static visuo-graviceptive conflict from body orientation. While we replicated enhanced self-identification with the seen body during synchronous as compared to asynchronous visuo-tactile stroking in the upright position (6), this difference was no longer significant in the tilted condition, as shown in questions tapping into body ownership and disembodiment.

Attenuation of illusory ownership due to an additional static visuo-proprioceptive conflict has been extensively studied in the rubber hand illusion. While the rubber hand is typically placed 10–15 cm to the side of the participant's hand, studies have presented the rubber hand rotated in yaw, e.g., 10–30° (25), with respect to the real hand. These data generally show a weaker illusion for increasing angles (30), especially if the rubber hand is rotated to an anatomically implausible position [angle of 135°, 180°, and 225° to the real hand; see Ref. (31)]. Yet, differences have been found between implicit and explicit measures regarding tolerance to this mismatch. Holle et al. (30), for example, showed a significant proprioceptive drift toward the rubber hand (implicit measure), while the questionnaire (explicit measure) suggested no illusion for a rubber hand rotated by 180°.

The attenuation of the full-body illusion reported here might be in line with these findings and importantly extends them from a body-centered toward a gravity-centered reference frame (32). On a more conceptual level, the attenuation of the illusion could be explained either by an influence of top-down knowledge about the body—e.g., anatomical plausibility or prior knowledge about body posture (31, 33)—or additional multisensory mismatches in the bottom-up process. This latter view is supported by data from the rubber hand illusion suggesting that even a slight angular mismatch, i.e., 10–30° rotation of the rubber hand, reduces illusory ownership over the rubber hand (25).

In contrast to previous studies (4, 16, 19), skin temperature, as an implicit measure of self-identification, was not significantly modulated by visuo-tactile synchrony. The validity of skin temperature as an index of self-identification with an external body has been debated (34), and null results have been found in several related studies (35, 36). This null finding stresses the need for other, more appropriate implicit measures, such as vertical perception.

### Vertical and Body Orientation Perception During the Full-Body Illusion

A main aim of this study was to test whether self-identification with a body that is tilted in relation to gravity would alter subjective haptic vertical perception and subjective body orientation perception [see Ref. (18) for a similar approach from a first-person perspective]. During illusory self-identification with the seen body in the tilted condition, we expected participants to align their subjective body orientation to the seen body. As a consequence, we expected them to adapt the perceived verticality by overcompensating (21). Both measures could serve as a useful implicit measure of the illusion (see above).

However, in the overall sample, we did not find a significant effect of Condition on haptic vertical perception. There are two possible reasons for the lack of a main effect of synchrony, which cannot be disentangled by the current protocol. First, the illusion in the tilted conditions may have been too weak to have a significant influence on haptic vertical and subjective body orientation perception. Indirect evidence for this hypothesis comes from our findings that visual-field-dependent participants actually do show a modulation of the haptic body orientation and verticality judgment (see below). Alternatively, the results could suggest that the measure is not sensitive to this modulation, which could be due to a very accurate gravity representation in an upright position (21) or a general strong role of non-visual signals on gravity perception, especially in the context of own-body perception. Yet, there are both physiological and behavioral measures showing that visual signals might overrule other graviceptive ones (37). Furthermore, against this hypothesis, we found a main effect of tilt on subjective body orientation, with the feeling of being more tilted toward the left, irrespective of visuo-tactile synchrony, which is in line with literature suggesting that looking at a tilted room alters perceived self-orientation (38).

### Effect of Visual Field Dependence/Independence on Visuo-Vestibulo-Tactile Integration

It is long known that individuals differ in the weight they put on various sensory systems during multisensory integration tasks, such as the rod-and-frame test (27). As expected, we found that visual field dependence influences perceived body orientation as a function of the synchrony of the stroking. Visual-field-dependent participants adapted their subjective body orientation more in the direction of the seen body in the synchronous than in the asynchronous visuo-tactile stroking condition. Furthermore, they adapted the subjective haptic vertical in the same direction. Although this result has to be interpreted with caution due to the small sample size, it suggests that visual field dependence influences implicit (but not the explicit) measures of the illusion, in line with previous literature (39, 40). Several studies showed that visual field dependence modulates illusory body perceptions. David et al. (41), for example, found a significant positive correlation between visual field dependence and proprioceptive drift in the rubber hand illusion. Moreover, visual field dependence was a good predictor of the perceived first-person perspective in a full-body illusion (17).

Our results show a selective adaptation of body orientation and verticality perception for visual-field-dependent individuals. These individuals showed stronger adaptation of the perceived body orientation during synchronous visuo-tactile stroking. Such adaptation of body perception to reduce the multisensory conflict could go in two directions: either participants perceive the visual body as closer to their own graviceptive reference (i.e., less tilted, which might be indicated by our findings in the questionnaire suggesting a stronger sensation of tilt in the asynchronous condition) or they perceive their own-body orientation as closer to the visual body (i.e., more tilted in line with our initial hypothesis). The fact that participants, irrespective of the type of stroking, adapted their body orientation to the seen body and room might give further evidence to the former hypothesis.

## Conclusions and Limitations of the Study

This study showed an attenuation of the full-body illusion during visuo-graviceptive conflict, providing empirical evidence for the importance of vestibular and other graviceptive cues in the moment-to-moment construction of our sense of a bodily self. The fact that only visual-field-dependent participants adapted the perceived body and gravity orientation to the seen and synchronously stroked body, further demonstrates the importance of individual weighting of sensory input in defining our bodily self. Future studies should further investigate such mutual interactions between body orientation in space and illusory self-identification. Since a 30° tilt in our study diminished the illusion, future studies should look at smaller orientation mismatches to be able to define the threshold and describe the effect of illusory tilt in the full sample. Furthermore, a limitation of our study was that we manipulated the orientation of the seen body and its surroundings. Future studies should try to disentangle the influence of the room tilt and the body tilt by rotating the two independently. Finally, it would be interesting to change the participant's actual orientation in space. It has been shown that verticality perception is less accurate (21) in positions different from upright, and illusory self-orientation and position in the room are more frequent in tilted positions in healthy participants and in epileptic and otoneurological patients (42, 43). Such manipulation would further allow inducing uncertainty in the prior belief about the participant's body orientation.

## Ethics Statement

This study was carried out in accordance with the recommendations of Ethics Committee of the Faculty of Arts and Social Sciences at the University of Zurich with written informed consent from all subjects. All subjects gave written informed consent in accordance with the Declaration of Helsinki. The protocol was approved by the Ethics Committee of the Faculty of Arts and Social Sciences at the University of Zurich.

## Author Contributions

CT, MR, and BL designed the experiment. CT, MR, and CB programmed the experiment. CT recorded the data. CT and BL analyzed the data. CT and BL drafted the manuscript. All authors reviewed the manuscript.

### Conflict of Interest Statement

The authors declare that the research was conducted in the absence of any commercial or financial relationships that could be construed as a potential conflict of interest.
