# Supplementary material for: The Tilted Self: Visuo-Graviceptive Mismatch in the Full-Body Illusion
Source: Front Neurol. 2019 May 8;10:436. doi: 10.3389/fneur.2019.00436 (PMC6517513; doi:10.3389/fneur.2019.00436)
Supplement: Supplementary file 1 [file Table_1.DOCX]

**Supplementary online material**

**Data recording and processing of the head tracking**

During the stroking period, head rotations in pitch (around the interaural axis), yaw (around the vertical axis) and roll (around the anterior-posterior axis) were recorded at 10 Hz using the head-tracking system of the HMD. The inertial measuring unit of the HMD consists of an accelerometer, a gyroscope and a magnetometer updating at 1000 Hz. Additionally, an external infrared camera tracked the position of the HMD in 6 degrees of freedom.

We calculated the average angle (as a measure of mean tilt) and standard deviation (as a measure movement amplitude over time) over the first 70 seconds for the pitch, yaw and roll axes.

**Results of the head-tracking**

The Friedman test showed a significant effect of Condition on the average head orientation in the yaw (χ^2^ = 14.8, p = .002) and roll (χ^2^ = 18.6, p < .001) planes, but not in the pitch plane (χ^2^ = 2.3, p = .52). Post hoc comparisons (Table S1) showed in yaw a significant larger rotation of the head to the left in the tilted condition compared to the non-tilted condition, for both synchronous (p = .006) and asynchronous (p < .001) visuo-tactile stroking. In roll, there was also a stronger tilt of the head to the left for the tilted condition compared to the non-tilted condition, for the asynchronous (p < .001) condition.

The Friedman test showed no significant effect of Condition on the standard deviation of the head tilt in the pitch (χ^2^ = 6.35, p < .09), yaw (χ^2^ = 3.24, p < .36) and roll (χ^2^ = 2.82, p < .42) planes.

| **Table S1** | **Friedman-Test** | **NT (S vs. AS)**  **(Illusion-effect upright)** | **T (S vs. AS)**  **(Illusion-effect tilt)** | **S (NT vs. T)**  **(Tilt-effect synchronous)** | **AS (NT vs. T)**  **(Tilt-effect**  **asynchronous)** |
| --- | --- | --- | --- | --- | --- |
| pitch | χ^2^ = 2.3 p = .52 |  |  |  |  |
| yaw | χ^2^ = 14.8 p = .002* | p = .74 | p = .66 | p = .006 * | p < .001 * |
| roll | χ^2^ = 18.6 p < .001* | p = .22 | p = .65 | p = .04 | p < .001 * |

**Table S1**: The table shows the results of the Friedman Test for the head tracking and post hoc Wilcoxon comparisons for the significant effects of the head tracking. * indicates significance level. For Friedman-Tests it was set to 0.05 and for the post hoc tests it was set to p=0.0125 according to the Bonferroni correction. NT = non-tilted, T = tilted, S = synchronous stroking, AS = asynchronous stroking.
